# Associations of Food Addiction Symptomatology and Disordered Eating Behaviors in a Pre-Surgical Bariatric Population

**DOI:** 10.3390/nu15153474

**Published:** 2023-08-06

**Authors:** Melissa Butt, Paddy Ssentongo, Ann M. Rogers, Andrea Rigby

**Affiliations:** 1 Department of Family and Community Medicine, Penn State College of Medicine, Hershey, PA 17033, USA; 2 Department of Public Health Sciences, Penn State College of Medicine, Hershey, PA 17033, USA; pssentongo@pennstatehealth.psu.edu (P.S.); arigby@pennstatehealth.psu.edu (A.R.); 3 Department of Medicine, Penn State Milton S. Hershey Medical Center, Hershey, PA 17033, USA; 4 Division of Minimally Invasive Surgery, Department of Surgery, Penn State Milton S. Hershey Medical Center, Hershey, PA 17033, USA; arogers@pennstatehealth.psu.edu

**Keywords:** food addiction, food insecurity, mental health, surgical attrition, surgical weight loss, bariatric surgery

## Abstract

The construct of food addiction (FA) has been highly debated in recent years particularly in the fields of disordered eating, medical weight management, and bariatric surgery. Some researchers have argued that FA symptoms are distinct, highly prevalent, and present a barrier for patients seeking medical treatment for obesity. The purpose of this study is to evaluate the cross-sectional associations between FA symptomatology, binge eating disorder (BED) and other appetitive traits, as well as dietary quality in a sample of adults with obesity seeking bariatric surgery. This post hoc analysis was conducted on a prospectively collected dataset from August 2020 to August 2022 at a single academic medical center. Descriptive statistics were used to characterize the sample. Additional analyses included: correlation coefficients, multivariable linear regression, and analysis of variance. A total of 587 patients were included in this analysis with low average scores for FA symptoms (mean: 1.48; standard deviation (SD): 2.15). Those with no BED symptoms had the lowest average FA symptoms scores (mean: 0.87; SD: 1.52) and those with both bingeing and LOCE had the highest average scores (mean: 3.35; SD: 2.81). This finding supports the hypothesis that, while related, FA and BED may represent different cognitions and behaviors.

## 1. Introduction

The construct of food addiction (FA) has been highly debated in recent years particularly in the fields of disordered eating, medical weight management, and bariatric surgery [1,2,3,4]. Some researchers have argued that FA symptoms are distinct, highly prevalent, and present a barrier for patients seeking medical treatment for obesity [5,6]. Others have argued that the symptoms of FA closely align with existing eating disorder behaviors such as binge eating, and challenge whether FA resembles a substance, rather than behavioral, addiction [7,8].

In recent years, there have been a number of advances in research aimed toward understanding FA in the medical literature. It is well known that taste receptors on the tongue respond strongly to salty and sweet foods [9]—foods that are considered highly palatable with low dietary quality [10]. Further, since the introduction and approval of glucagon-like peptide-1 (GLP-1) agonists for the treatment of diabetes and obesity, there is new evidence that these medications can disrupt neurohormonal signals, thereby leading to hunger control, as well as diminished desire for highly palatable foods [11]. While much of the bench research shows promise for identifying the mechanisms behind what we currently understand of FA [12,13], the behavioral understandings are less clear.

In 2009, researchers at Yale University developed the first patient-reported outcome measure based on clinical criteria of substance-based addictions [14]. This was updated in 2016 following the publication of and updates to the Diagnostic and Statistical Manual of Mental Disorders—Fifth Edition (DSM-V) on substance-related addictive behaviors [15]. While these criteria have been used in research to evaluate patients for FA symptomatology, there is still no formal diagnosis that clinicians can use to guide treatment, unlike other substance use disorders and behavioral addictions such as gambling.

In 2013, the DSM-V included the diagnosis of binge eating disorder (BED) as a formal eating disorder characterized by consuming large amounts of food in a specified time period and feeling a sense of loss of control during that eating episode [16]. Binge eating disorder is the most prevalent of the eating disorders [16]. In addition to BED, additional disordered eating behaviors have previously been identified, such as night eating syndrome and purging, both of which share some clinical associations with food addictive behavior [17,18,19,20,21].

While some overlap between FA and other disordered eating behaviors may exist, the extent of these associations has not been well studied, particularly in a presurgical bariatric population. Consequently, the purpose of this study is to evaluate the cross-sectional associations between FA symptomatology, BED and other disordered eating behaviors, as well as dietary quality in a sample of treatment-seeking adults with obesity. The authors hypothesized that there would be significant yet minor associations between those who disclosed more symptoms of FA and the other outcomes of interest.

## 2. Materials and Methods

This post hoc analysis was conducted on a prospectively collected dataset of adults seeking bariatric surgery from August 2020 to August 2022 at a single academic medical center. This center is nationally accredited through the Metabolic and Bariatric Surgery Accreditation and Quality Improvement Program (an arm of the American College of Surgeons) and provides care to patients across the south-central Pennsylvania region. Upon entry into the program and prior to surgery, patients undergo psychological testing as a part of the standard process for preparation for surgery. This testing occurs around the 1-month mark of the patients’ per-surgical process after an initial clinical screening process that confirms their eligibility for the surgery and insurance coverage. The testing was administered via REDCap, a secure, online, web-based application [22]. A link for the test was sent to the patient to complete individually and reviewed by the clinical psychologist as a part of the standard psychological evaluation process for this site. De-identified data from adult patients were extracted, and a post hoc analysis was then conducted to evaluate the associations between the variables of interest. This study was reviewed and approved by the Penn State University Institutional Review Board.

### 2.1. Measures

#### 2.1.1. Food Addiction Symptomatology

The primary outcome variable of interest was FA as measured by the modified Yale Food Addiction Scale 2.0 (mYFAS) [23]. This instrument consists of 13 questions with a frequency-based response option ranging from never (“0”) to every day (“7”). Each item is assigned a frequency threshold, and if the response surpasses that threshold, the item is recorded as “1”. The recorded values for the 11 symptom-based items are then summed for a total score range of 0 to 11. The remaining two items were used to assess clinical significance. For the purposes of this analysis, only the symptom scores were used in the models, with the clinical significance items being assessed discretely in four categories: no clinical significance, distress only, impairment only, and distress and impairment. The internal consistency of the FA symptoms scores in this sample were strong, with a standardized Cronbach’s α of 0.80.

#### 2.1.2. Eating Behaviors

Eating behaviors were measured using multiple instruments: the Adult Eating Behavior Questionnaire (AEBQ) [24] and the two binge eating criteria screening questions from the Weight and Lifestyle Inventory (WALI) [25]. Additionally, dietary quality was included in this analysis and measured using the Rapid Eating Assessment for Participants—Short Form (REAP-S) [26]. The AEBQ is a previously validated 35-item instrument [27] that measures eight different appetitive traits divided into two categories: food approach and food avoidant traits. Food approach traits include enjoyment of food, emotional overeating, food responsiveness, and hunger, while food avoidant traits include satiety responsiveness, emotional undereating, slowness in eating, and food fussiness. Each category has 3, 4 or 5 items measured using a 5-point Likert scale and summed together for a total score. Binge eating symptomatology was divided into four categories based on the screening questions from the WALI: no symptoms, bingeing only, loss of controlled eating (LOCE) only, and both bingeing and LOCE. The first 13 items of the REAP-S were used to assess dietary quality. These items were measured using a 3-point Likert scale with a total score range of 13 (low dietary quality) to 39 (high dietary quality). The internal consistency of the REAP-S was adequate, with a Cronbach’s α of 0.65.

#### 2.1.3. Additional Measures

Sociodemographic variables including age, sex, race (categorized as White and Non-White), and highest level of educational attainment were collected from the patients. Participants were also asked to complete the Marlow Crowne Social Desirability Scale 13-item Short Form (MC-C) [28], the Beck Depression Inventory II (BDI-II) [29], and the Burns Anxiety Inventory (BAI) [30]. Patients were also asked to self-report their height and weight, which were then used to calculate body mass index (BMI; kilogram/square meter).

### 2.2. Statistical Analysis

These data were analyzed in April of 2023 using SAS Version 9.4 (SAS Institute, Cary, NC, USA) with the level of significance set to 0.05. Due to the self-reported nature of the testing process, there were some missing data for each of the variables, identified in the corresponding tables. All scores were calculated as instructed. Due to the nonparametric distribution of the BDI-II and BAI scores, these instruments were used in their ordinal scales as opposed to their continuous scores. Continuous variables were standardized using the total sample standard deviation of the instrument when included in models as independent variables.

Descriptive statistics were used to characterize the sample characteristics and item scores. Univariable associations were assessed using Spearman Correlation Coefficients with 95% Confidence Limits (CL) and linear regression models with standardized independent variables. Spearman correlation coefficients of 0.10 to 0.39 were considered weak and 0.40 to 0.69 were considered moderate [31]. These models were also adjusted for socially desirable responding in order to account for possible impression management. Two multivariable linear regression models were built using FA symptomatology as the dependent variable and disordered eating behaviors as the independent variables. One of these models included all of the variables of interest while the second employed stepwise selection methods to obtain a reduced model. The significance level for entry and stay was set to 0.15. Both models included socially desirable responding as a control variable and goodness of fit was assessed using the Akaike information criterion (AIC) and adjusted R2 values. Known groups validity was also assessed between FA symptoms and binge eating criteria categories using analysis of variance (ANOVA) for unadjusted models and analysis of covariance (ANCOVA) for adjusted models, controlling for socially desirable responding. Pairwise comparisons for the ANOVA models also included Tukey adjustments for multiple comparisons. There was no significant association between FA symptomatology and age, sex, or BMI in the univariable associations, so multivariable regression models were not controlled for these variables.

## 3. Results

A total of 587 patients were included in this analysis (Table 1). The mean (μ) age of the sample was 43 years (standard deviation (SD): 11.74), and most patients were female (*n* = 457, 77.99%), identified as White (*n* = 432; 74.23%), and had an educational attainment of high school or less (*n* = 287; 49.91%). Socially desirable responding scores were high with a mean of 9.37 (SD: 2.56). Mental health screeners revealed minimal rates of adverse psychopathology with 63.06% (*n* = 367) disclosing no depressive symptoms and 52.69% (*n* = 304) disclosing mild or no anxious symptoms at the time of screening.

In terms of disordered eating (Table 2), dietary quality scores were moderate with a mean of 28.32 (SD: 4.02) and low average scores for FA symptomatology (μ: 1.48; SD: 2.15). The majority of participants indicated their FA symptoms (if any) had no clinical significance (*n* = 455; 79.55%), while 28 (4.90%) indicated distress, 42 (7.34%) indicated impairment, and 47 (8.22%) indicated both distress and impairment. The majority of participants also disclosed no binge eating disorder symptoms (*n* = 351; 60.10%), while 92 (15.75%) indicated bingeing only, 37 (6.34%) indicated LOCE only, and 104 (17.81%) indicated both. Proportional to the total possible score, participants scored higher on food approach behaviors such as enjoyment of food (μ: 11.58; SD: 2.17) and food responsiveness (μ: 11.26; SD: 3.21) than food avoidant behaviors such as food fussiness (μ: 12.29; SD: 4.41).

Univariably (Table 3), FA symptom scores were significantly associated with all variables of interest. There was a weak inverse correlation between FA symptomatology and socially desirable responding (ρ = −0.38), satiety responsiveness (ρ = −0.25), and dietary quality (ρ = −0.21). Additionally, there was a weak correlation between FA symptoms and all food approach behaviors: enjoyment of food (ρ = 0.32) and hunger (ρ = 0.34) as well as a moderate correlation with emotional overeating (ρ = 0.44) and food responsiveness (ρ = 0.45). Further, there was a moderate correlation between FA symptoms and both anxious (ρ = 0.45) and depressive (ρ = 0.50) symptoms. The regression coefficients reflected similar patterns in associations with and without controlling for socially desirable responding. Most notably, the food approach behaviors saw significant increases in FA scores for food responsiveness (β = 0.81), emotional overeating (β = 0.79), hunger (β = 0.66), and enjoyment of food (β = 0.50) after controlling for socially desirable responding.

When accounting for all disordered eating variables of interest, two models were compared: an expanded and a reduced model (Table 4). After a stepwise selection of the variables, binge eating criteria (β = 0.45) and three of the four food approaches (emotional overeating [β = 0.30]; food responsiveness [β = 0.36], and hunger [β = 0.22]) were significantly associated with higher FA symptoms while emotional undereating (β = −0.24) was inversely associated with FA symptoms. Of the two models, the reduced model demonstrated better goodness of fit with a lower AIC and higher adjusted R2 value.

**Figure 1:**
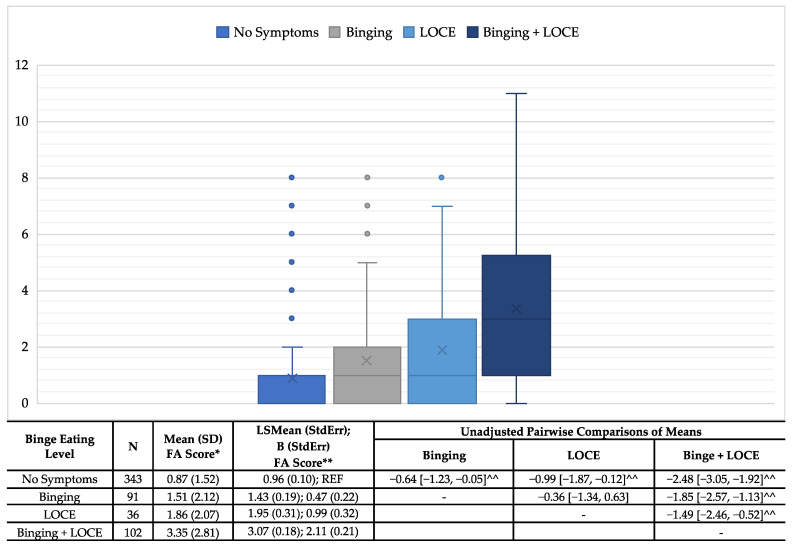
Associations of food addiction scores and binge eating criteria—known groups validity. *ANOVA: Pairwise comparisons included Tukey adjustment for multiple comparisons; **ANCOVA: controlling for socially desirable responding; ^^Unadjusted and adjusted comparisons significant.

The known groups validity between FA and binge eating symptoms showed a positive correlation, with an increasing severity of binge eating symptoms (Figure 1). Those with no symptoms had the lowest average FA symptoms scores (μ: 0.87; SD: 1.52) and those with both bingeing and LOCE had the highest average scores (μ: 3.35; SD: 2.81). Additionally, those with LOCE only had slightly higher mean scores (μ: 1.86; SD: 2.07) than those who reported bingeing only (μ: 1.51; SD: 2.12). Additionally, all pairwise comparisons were significant with the exception of bingeing only to LOCE only. These trends remained the same after controlling for socially desirable responding.

## 4. Discussion

The current study sought to evaluate the associations between FA symptomatology and eating behaviors in a pre-surgical bariatric program as well as to assess known groups validity with binge eating disorder criteria. A number of studies have been conducted in recent years examining the phenomenon of FA among those seeking bariatric surgery [5,6], especially given the association between FA and overweight/obesity [32,33]. While the average rates of FA symptoms were arguably low in this particular study, the total number of symptoms ranged from 0 to 11 out of the 11 possible symptoms. A 2017 review identified 10 studies that evaluated FA in a pre-surgical bariatric population, with FA rates ranging from 14% to 58% [5]; however, these studies predominantly used the original Yale Food Addiction Scale as opposed to the modified second edition used herein.

This study also found moderate bivariate associations between FA and depression and anxiety. Witek et al. highlighted in a recent review that diets high in sucrose can lead to increases in depressive and anxious symptoms [34]. Another recent study found significant associations between FA and depression and anxiety in a sample of university students [35]. This finding is not alarming given previous reports of the use of food as a coping method, to comfort oneself in a time of stress, or in response to strong emotions across a number of different populations [36,37,38,39]. One important factor to note is that this particular analysis is cross-sectional in nature; thus, it is impossible to make assumptions on the directionality of these associations within this specific population.

Moreover, this study found that FA symptoms were inversely correlated with dietary quality. In other words, FA symptoms were lower among those with higher dietary quality scores. The authors were not able to identify additional studies in the literature that evaluated the associations between FA symptoms and dietary quality. Previous studies have identified that dietary quality is highly variable in a pre-surgical bariatric patient population [40]. Additionally, poorer dietary quality is further associated with an increased weight status [41]. Identifying a connection between dietary quality and FA is not a leap, as research demonstrates that poor dietary quality (especially among US populations) largely comprises foods high in fats, sugars, and salts [34]. Additionally, in a recent systematic review of 16 studies, FA was associated with a higher intake of sugar, processed/energy-dense foods, total fat, and proteins for those with overweight/obesity when compared to the general population [42]. Given the palatability of these foods and previously identified addictive qualities of foods high in sugar, it would be reasonable to assume that poorer dietary quality would be associated with increased FA symptomatology.

A number of studies have previously identified the associations between FA and maladaptive eating behaviors [19,32,33,43]. Our findings are consistent with trends in the current literature. More broadly, a recent study by Echeverri et al. found significant associations between night eating syndrome and FA symptoms in a general sample of 166 adults [19]. Another study in 2014 utilized the Eating Disorder Examination Questionnaire and found higher rates of disordered eating pathology among those with higher FA scores [32]. Additionally, a recent study found higher levels of grazing behaviors among those with moderate to severe FA scores in a sample of individuals with overweight or obesity [44].

Specifically, the current study found a positive correlation between FA symptoms and food approach eating behaviors as well as an inverse correlation with food avoidant behaviors. In particular, our study demonstrated that food approach eating behaviors (i.e., food responsiveness, emotional overeating, hunger and enjoyment of food) were most highly correlated with FA symptoms in our univariable models and many remained significantly associated in the multivariable models. A recent study conducted within a sample of university students in Spain utilized the Dutch Eating Behavior Questionnaire (DEBQ) evaluating emotional, external, and restrained eating, and found significant correlations between FA symptoms and all three subscales [43]. Additional studies found a similar positive association between FA symptoms and emotional and external eating [45,46,47].

The inclusion of food avoidant eating behaviors (i.e., satiety responsiveness, emotional undereating, food fussiness and slowness in eating) in the current study is quite unique, but also consistent with the associations between the restrained eating subscale of the DEBQ. Previous studies have also shown the benefits and protective effects of promoting satiety [48,49,50]. Additionally, most current pharmacological interventions for medical weight management, such as the use of phentermine-topiramate combinations or GLP-1 agonists, work to suppress appetite and promote feelings of satiety in order to achieve weight loss [51,52,53]. A number of studies have previously highlighted the benefits of slowness in eating as a behavioral intervention to help reduce weight [54]. Given the inverse associations between these variables identified in the current sample, it may be worth investigating behavioral interventions to promote satiety when eating, as well as to encourage slower eating to combat symptoms of FA.

Further, this study identified a positive and increasing correlation between the number of FA symptoms and the BED criteria. These findings are consistent with the current literature, as a number of studies have identified a positive association between BED and FA symptoms including a general sample of Australian adults [55], a sample of Spanish university students [43], a general sample of American adults [32], a clinical sample of treatment-seeking patients with obesity [33], and a combined clinical and general population of Italian adults [56]. The current study differs from the available literature through its use of a sample of adult patients seeking bariatric surgery for the treatment of obesity. Further, this study builds upon the current literature by identifying the differences in the average number of FA symptoms with different known BED criteria. Specifically, this study identifies that endorsing no BED criteria was associated with the lowest average FA symptoms while those endorsing both BED criteria (i.e., bingeing and LOCE) had the highest average FA symptoms. It is important to note, however, that FA symptoms were not entirely absent among those who endorsed no BED criteria, further supporting the hypothesis that, while related, FA and BED may not be entirely predictive of each other. Additionally, those who endorsed both BED criteria had a significantly higher FA score than those with bingeing or LOCE alone. Interestingly, the average scores between those with bingeing or LOCE were not significantly different, suggesting that these elements by themselves are impacted by FA symptom pathology at similar rates.

### 4.1. Clinical Implications

This study adds to the body of literature regarding the construct of FA, and suggests that FA, while aligning closely with binge eating behaviors, may be a distinct and separate group of cognitions and behaviors. This finding has clinical implications in that treatment for FA may be focused on food approach behaviors such as food responsiveness, along with satiety responsiveness and slow eating. Additionally, treatment may be specifically tailored toward symptoms such as craving for highly palatable foods in the absence of consumption of large quantities of food as is the case with BED. Unlike other substance use disorders or behavioral addictions where abstinence from the trigger or substance is essential for treatment, the same cannot be said for those experiencing symptoms of FA. Tailoring treatment to target the addictive behaviors and tendencies prior to surgery could also provide a more structured approach to weight management following surgery to prevent relapse of previous eating behaviors and possible weight recurrence.

### 4.2. Strengths and Limitations

A number of limitations should be considered when interpreting the findings of the current study. First, this analysis only includes patients from a single academic medical center and seeking bariatric surgery. Further, it is important to note that these evaluations were collected from 2020 to 2022, after the holds on elective medical procedures were lifted following the COVID-19 pandemic. The impact of the COVID-19 pandemic on individual lives should not be understated and may result in historical bias within the findings. Consequently, the results may not be generalizable to a larger sample or a population/time period. Additionally, the cross-sectional nature of the study prevents the authors from making assumptions regarding the temporality or predictive abilities of the associations. Lastly, as evident by the high socially desirable responding scores, response bias is likely to have impacted the results.

Despite these limitations, the sample itself is considerably large and consistent with the national averages for those seeking bariatric surgery in terms of age and sex, with only slightly higher rates of white participants [57]. Additionally, the data had arguably low rates of missing data allowing for a more complete analysis. Further, as a measure of socially desirable responding was included in the evaluations, the authors were able to control for this variable in order to minimize the impact of impression management.

## 5. Conclusions

While previous studies have demonstrated associations between FA and BED, this is the first study to evaluate the known groups validity between the BED criteria and FA symptoms using a large clinical sample of patients seeking bariatric surgery. Most notably, symptoms of FA were not entirely absent among those who endorsed no BED criteria, in addition to the fact that a portion of those who endorsed both criteria (bingeing and LOCE) reported minimal to no FA symptoms. This finding supports the hypothesis that, while related, FA and BED may represent discrete cognitions and behaviors. Future studies are needed to identify the clinical importance of FA in the treatment of obesity as well as to establish clear clinical criteria for the diagnosis and treatment thereof. Additionally, future studies should consider how surgical selection and post-operative outcomes factor into these associations.

## Figures and Tables

**Table 1:** Sample characteristics (*n*= 587).

Variable	Total Sample	No. Missing
Body Mass Index—mean (SD)	48.08 (8.93)	10
Age—mean (SD)	42.97 (11.74)	2
MC-C—mean (SD)	9.37 (2.56)	12
Sex—*n* (%)		1
Male	129 (22.01)	
Female	457 (77.99)	
Ethnicity—*n* (%)		5
White	432 (74.23)	
Non-White	150 (25.77)	
Education—*n* (%)		12
HS or Less	287 (49.91)	
Some College	114 (19.83)	
Bachelors	113 (19.65)	
Graduate	61 (10.61)	
BDI—II—*n* (%)		5
None	367 (63.06)	
Minimal	92 (15.81)	
Moderate	81 (13.92)	
Severe	42 (7.33)	
BAI—*n* (%)		6
None	155 (26.68)	
Mild	149 (25.65)	
Borderline	130 (22.38)	
Moderate	66 (11.36)	
Severe	62 (10.67)	
Extreme	19 (3.27)	

MC-C: Marlow Crowne Social Desirability Scale 13-item Short Form; BAI: Burns Anxiety Inventory; BDI-II: Beck Depression Inventory—II; SD: standard deviation

**Table 2:** Dietary quality and eating behaviors.

Variable	Total Sample (*n* = 587)	Total Score Range	No. Missing
REAP-S—mean (SD)	28.32 (4.02)	13–39	2
mYFAS —Symptoms—mean (SD)	1.48 (2.15)	0–11	15
mYFAS —Clinical Significance—*n* (%)			15
No Clinical Significance	455 (79.55)		
Distress	28 (4.90)		
Impairment	42 (7.34)		
Distress AND Impairment	47 (8.22)		
Binge Eating—*n* (%)			3
No symptoms	351 (60.10)		
Binging only	92 (15.75)		
LOCE only	37 (6.34)		
Binging and LOCE	104 (17.81)		
AEBQ—mean (SD)			12
Enjoyment of Food	11.58 (2.17)	3–15	
Food Responsiveness	11.26 (3.21)	4–20	
Emotional Overeating	13.69 (5.06)	5–25	
Hunger	13.51 (3.64)	5–25	
Satiety Responsiveness	10.81 (2.94)	4–20	
Food Fussiness	12.29 (4.41)	5–25	
Slowness in Eating	10.64 (3.71)	4–20	
Emotional Undereating	13.35 (4.53)	5–25	

REAP-S: Rapid Eating Assessment for Participants—Short Form; mYFAS: Modified Yale Food Addiction Scale; AEBQ: Adult Eating Behavior Questionnaire; SD: Standard Deviation.

**Table 3:** Univariable associations with food addiction symptomatology scores (*n* = 572).

Variable	Spearman Correlation Coefficient (95% CL)	Unadjusted Linear Regression **	*p*-Value	Adjusted Linear Regression ***	*p*-Value
MC-C	−0.38 (−0.45, −0.31) *	−0.81 (0.08)	<0.0001	-	-
Satiety Responsiveness	−0.25 (−0.32, −0.17) *	−0.54 (0.09)	<0.0001	−0.38 (0.08)	<0.0001
REAP-S	−0.21 (−0.29, −0.13) *	−0.44 (0.09)	<0.0001	−0.27 (0.10)	0.0015
Slowness in Eating	−0.18 (−0.25, −0.10) *	−0.37 (0.09)	<0.0001	−0.25 (0.08)	0.003
Emotional Undereating	−0.15 (−0.23, −0.07) *	−0.38 (0.09)	<0.0001	−0.35 (0.08)	<0.0001
Food Fussiness	0.11 (0.02, 0.19) *	0.19 (0.09)	0.03	0.12 (0.08)	0.14
Enjoyment of Food	0.32 (0.24, 0.39) *	0.70 (0.08)	<0.0001	0.50 (0.08)	<0.0001
Hunger	0.34 (0.27, 0.41) *	0.82 (0.08)	<0.0001	0.66 (0.08)	<0.0001
Emotional Overeating	0.44 (0.38, 0.51) *	0.98 (0.08)	<0.0001	0.79 (0.08)	<0.0001
Food Responsiveness	0.45 (0.38, 0.51) *	1.00 (0.08)	<0.0001	0.81 (0.08)	<0.0001
BAI	0.45 (0.38, 0.51) *	0.66 (0.06)	<0.0001	0.53 (0.06)	<0.0001
BDI-II	0.50 (0.43, 0.56) *	1.14 (0.08)	<0.0001	0.97 (0.08)	<0.0001

MC-C: Marlow Crowne Social Desirability Scale 13-item Short Form; REAP-S: Rapid Eating Assessment for Participants—Short form; BAI: Burns Anxiety Inventory; BDI-II: Beck Depression Inventory—II; CL: Confidence Limits. * Significant *p* < 0.05. ** Presented as β coefficient (standard error); independent variable was standardized using the sample standard deviation. *** Presented as β coefficient (standard error); independent variable was standardized using the sample standard deviation and controlled for socially desirable responding.

**Table 4:** Linear regression model predicting food addictive symptomology (*n* = 572).

Variable	Expanded Model	Reduced *
Binge Eating Criteria	0.44 (0.07); <0.0001	0.45 (0.07); <0.0001
Food Responsiveness	0.34 (0.11); 0.002	0.36 (0.10); 0.0004
Emotional Overeating	0.31 (0.09); 0.0012	0.30 (0.09); 0.0013
Emotional Undereating	−0.24 (0.08); 0.002	−0.24 (0.08); 0.002
Hunger	0.22 (0.09); 0.02	0.22 (0.09); 0.02
Food Fussiness	0.11 (0.07); 0.12	0.11 (0.07); 0.11
Dietary Quality	−0.08 (0.08); 0.28	-
Enjoyment of Food	0.06 (0.09); 0.48	-
Satiety Responsiveness	0.03 (0.09); 0.76	-
Slowness in Eating	0.01 (0.08); 0.93	-
AIC	622.77	616.53
Adjusted R^2^	0.369	0.372

AIC: Akaike information criterion (AIC). Model estimates are presented as β coefficient (standard error); *p*-value. * Stepwise selection.

## Data Availability

The data presented in this study are available on request from the corresponding author. The data are not publicly available due to regulatory and privacy concerns.
